# Case of a Woman Delivered of Five Children

**Published:** 1840-01

**Authors:** 


### *Singular Case of a Woman delivered of Five Children.*

Giuseppa Califani, of Naples, at the age of fourteen years and three months, was married to a man aged twenty-seven, by whom she had ten children at eight accouchements; at the fifth and sixth producing twins. She lived with her husband ten years, and remained a widow three years after his death; she then took a second husband, whose age was about twenty-nine. After two regular accouchements upon her third pregnancy she became enormously large; so that, at seven months she appeared to be at the termination of her natural period. She was taken, however, at seven months, with labor-pains, and brought forth, successively, and by natural presentation, five living children, all of whom were baptized. The mother did not suffer anything extraordinary. Four of these children were females, and one male. The male infant was delivered first, and after a few minutes one female; then after a cessation of fifteen minutes' interval between each, the other three followed. The infants much resembled each other, and were of a regular form and well grown, and very nearly of the ordinary size of a seven months' fœtus; each weighed about 3½lbs., and measured in length a French foot. The insertion of the umbilical cord was about four lines lower down than ordinarily. The placentas with their membranes were four instead of five; and each had its proper umbilical cord, except the fourth, which contained two in one large sac. The fœtus, with their membranes, placenta, and umbilical cords, are preserved in the Royal Anatomical Museum of the University of Naples.—*Bulletine delle Scinze Mediche.*

